# Homeostatic synaptic scaling: molecular regulators of synaptic AMPA-type glutamate receptors

**DOI:** 10.12688/f1000research.13561.1

**Published:** 2018-02-28

**Authors:** Dhrubajyoti Chowdhury, Johannes W Hell

**Affiliations:** 1 Department of Pharmacology, University of California Davis, Davis, California, USA

**Keywords:** synapse, glutamate receptors, AMPARs, homeostatic plasticity

## Abstract

The ability of neurons and circuits to maintain their excitability and activity levels within the appropriate dynamic range by homeostatic mechanisms is fundamental for brain function. Neuronal hyperactivity, for instance, could cause seizures.  One such homeostatic process is synaptic scaling, also known as synaptic homeostasis. It involves a negative feedback process by which neurons adjust (scale) their postsynaptic strength over their whole synapse population to compensate for increased or decreased overall input thereby preventing neuronal hyper- or hypoactivity that could otherwise result in neuronal network dysfunction. While synaptic scaling is well-established and critical, our understanding of the underlying molecular mechanisms is still in its infancy. Homeostatic adaptation of synaptic strength is achieved through upregulation (upscaling) or downregulation (downscaling) of the functional availability of AMPA-type glutamate receptors (AMPARs) at postsynaptic sites.  Understanding how synaptic AMPARs are modulated in response to alterations in overall neuronal activity is essential to gain valuable insights into how neuronal networks adapt to changes in their environment, as well as the genesis of an array of neurological disorders. Here we discuss the key molecular mechanisms that have been implicated in tuning the synaptic abundance of postsynaptic AMPARs in order to maintain synaptic homeostasis.

## Introduction

Homeostatic synaptic scaling allows neurons to maintain their activity within a dynamic range despite ongoing alterations in input activity, thereby ensuring normal propagation of signal through the neuronal network. For instance, synaptic modifications underlying memory formation are inherently destabilizing because of the positive feedback nature of the cellular correlates, long-term potentiation (LTP) and long-term depression (LTD), and necessitate the existence of homeostatic adaptations to normalize overall synaptic strength. Homeostatic regulation of synaptic strength preserves the excitation/inhibition balance in the brain, which is critical for proper brain functions and often dysregulated in several neurological disorders ^1^. Synaptic scaling of excitatory synaptic transmission is a negative feedback process involving adjustment in post-synaptic strength to compensate for input activity. Such a phenomenon was initially described, about 20 years ago, in dissociated neuronal cultures upon pharmacological manipulation of neuronal activity. Accordingly, chronic silencing by the voltage-gated sodium channel blocker tetrodotoxin (TTX) resulted in upregulation (upscaling) of synaptic strength ^2, 3^. Chronic elevation of neuronal activity by the GABAA receptor antagonist bicuculline (Bic) caused downregulation (downscaling) of synaptic strength. These alterations in synaptic strength are observed as an increase or decrease in the amplitude of miniature excitatory post-synaptic current (mEPSC) mediated by AMPA-type glutamate receptors (AMPARs), which account for the majority of fast excitatory transmission in the central nervous system. Bidirectional scaling of AMPAR–mEPSC amplitude in principal neurons of rat primary visual cortex in response to changes in visual experience of the animal was demonstrated thereafter ^4^, thus establishing the existence of this phenomenon *in vivo*. Such functional changes are the consequences of altered synaptic AMPAR content, which is regulated by diverse molecular mechanisms, and these form the theme of this article. Apart from the homeostatic adaptation in excitatory post-synaptic strength, other processes such as change in pre-synaptic neurotransmitter release ^5^, change in intrinsic excitability ^6^, and scaling of inhibitory synapses ^7^ are known to stabilize neuronal activity.

## Molecular mechanisms

The molecular mechanisms underlying synaptic scaling of excitatory synapses converge upon the regulation of synaptic AMPARs in terms of number or subunit composition or both. AMPARs are hetero-tetrameric glutamate-gated ion channels assembled as a dimer of dimers comprising GluA1–GluA4 subunits ^8^. Subunit composition dictates key channel properties, including agonist affinity, gating kinetics, and calcium permeability, and subunit switching offers an effective means to alter synaptic transmission without changing receptor number. AMPARs are highly mobile, and their synaptic abundance is tightly regulated by a host of mechanisms that impact their trafficking to/from the synapse, mobility along the plasma membrane, and stabilization at the post-synaptic density (PSD) ^9^. Such mechanisms include a variety of post-translational modifications of the receptor itself as well as post-synaptic scaffolding and accessory proteins that interact with AMPARs at specific steps of the receptor’s life cycle. In this review, we highlight some of the key molecular regulators of synaptic AMPARs that have been implicated in synaptic scaling.

### Phosphorylation of GluA1 on Ser845

Phosphorylation of Ser845 on the GluA1 subunit of AMPARs by protein kinase A (PKA) promotes the insertion of AMPARs into the cell surface ^10– 13^ and especially in the perisynaptic space ^14– 16^. This mechanism augments the accumulation of AMPARs at post-synaptic sites upon the induction of LTP ^10, 17^. Dephosphorylation of Ser845 by the calcium-dependent phosphatase calcineurin is correlated with endocytosis of AMPARs ^18^, which is necessary for LTD ^19– 21^. In addition, this phosphorylation increases channel open probability and hence potentiates individual receptor function ^22^. GluA1–Ser845 phosphorylation within the PSD is increased during TTX-induced upscaling and decreased upon Bic-induced downscaling in cultured neurons ^23^. Such bidirectional change in GluA1–Ser845 phosphorylation matches the enhanced and reduced AMPAR synaptic levels in upscaling and downscaling, respectively, although only TTX-induced scaling up was impaired in neurons from S845A GluA1 knock-in mice. Moreover, the upregulation of AKAP5-anchored PKA activity was suggested to drive scaling up ^23^. In another study using dissociated cultures, reduced activity of calcineurin was found to underlie upscaling mediated by Ca ^2+^-permeable AMPARs (CP-AMPARs) via increased GluA1–Ser845 phosphorylation ^24^. Moreover, increased Ser845 phosphorylation is correlated with and necessary for AMPAR–mEPSC scaling up in visual cortex following visual deprivation ^25, 26^. This finding highlights its importance in *in vivo* sensory experience-induced synaptic upscaling. However, enhanced GluA1–Ser845 phosphorylation is not sufficient for producing multiplicative scaling in this system, indicating requirements for additional events for synaptic recruitment of AMPARs. We propose that Ser845 phosphorylation promotes post-synaptic AMPAR accumulation during homeostatic synaptic upscaling. How the underlying chronic decrease in synaptic input induced Ser845 phosphorylation is not known but could be due to a reduction in the activity of the phosphatase that dephosphorylates Ser845. As discussed in the next section, GluA1-containing AMPARs have been widely implicated in synaptic scaling ^27– 29^. These findings are consistent with a role of Ser845 phosphorylation in synaptic scaling up. Furthermore, GluA1 can form homo-tetrameric CP-AMPARs, which have been implicated in scaling up. That those GluA1 homomers possess four Ser845 phosphorylation sites makes them perhaps especially suitable for contributing to scaling up.

### Role of Ca ^2+^-permeable versus Ca ^2+^-impermeable AMPARs in synaptic scaling

Despite the implication of GluA1-containing AMPARs in synaptic scaling ^27– 29^, there is an ongoing debate regarding the requirement for GluA2-containing AMPARs, particularly in upscaling. A switch from Ca ^2+^-impermeable GluA2-containing AMPARs (CI-AMPARs) to higher-conductance CP-AMPARs at the synapse can have significant consequences for intracellular signaling, which might contribute to homeostatic upscaling. Such subunit switch will serve the purpose of homeostatic excitatory synaptic gain in a rapid and efficient manner. Chronic TTX treatment of an organotypic hippocampal slice preparation leads to selective upregulation of GluA2-lacking AMPARs in CA1 pyramidal neurons ^30^. Similarly, a 4-hour treatment with TTX with additional NMDA-type glutamate receptor (NMDAR) blockade with 2-amino-5-phosphonopentanoic acid (APV) during the last 1–3 hours induces temporary insertion of CP-AMPARs at post-synaptic sites ^31^. Chronic blockade of either AMPARs or L-type Ca ^2+^ channels also induces post-synaptic localization and response of CP-AMPARs in dissociated hippocampal cultures ^27^. These findings clearly implicate at least temporary post-synaptic functional appearance of CP-AMPARs in synaptic upscaling, which is reminiscent of a somewhat analogous temporary post-synaptic appearance of CP-AMPARs during LTP in very young (2-week-old) as well as more-than-7-week-old mice but not in 3- to 4-week-old mice ^21, 32– 34^. Furthermore, microRNA-mediated selective repression of GluA1 and GluA2 expression in dissociated hippocampal neurons prevents and facilitates upscaling, respectively, by chronic TTX/APV treatment ^35, 36^, further suggesting the replacement of CI-AMPARs by CP-AMPARs in upscaling. However, both GluA1 expression and GluA2 expression are affected upon treatment with TTX alone ^2, 37, 38^, indicating divergent mechanisms operative in response to the suppression of action potential with or without NMDAR signaling. Interestingly, TTX-induced upscaling is preserved in hippocampal cultures from constitutive GluA1 or GluA2 knockout (KO) mice ^39^, indicating that upscaling can be achieved simply by increasing the number of AMPARs by recruitment and retention mechanisms that bypass subunit-specific interactions and rather modulate receptor trafficking and post-synaptic organization. However, developmental compensation could account for these effects using this approach. In fact, knockdown of GluA2 and ectopic expression of the cytosolic GluA2 C-terminus, which interacts with glutamate receptor-interacting protein 1 (GRIP1), protein interacting with C-kinase 1 (PICK1), NSF, and AP2, impair upscaling in response to chronic TTX treatment in dissociated cortical neurons ^37^. Accordingly, GluA2-containing AMPARs are also required for upscaling in wild-type animals. These findings are consistent with the requirement of GRIP1 and PICK1 in upscaling (see next paragraph) ^38, 40, 41^. A more recent study measuring asynchronous EPSCs (aEPSCs) in response to evoked inputs in hippocampal slice culture, where either GluA1 or GluA2 was knocked down in sparse CA1 pyramidal neurons, concluded that GluA2, not GluA1, is necessary for TTX-induced scaling ^42^. Furthermore, GluA2 is found to be sufficient for scaling based on molecular replacement of endogenous GluA1–3 with the unedited rectifying GluA2 (Q) subunit, although all of these genetic manipulations reduced baseline aEPSC amplitude. Obviously, GluA1 and GluA2 are important for scaling up, and the relative contributions might vary depending on exact conditions and systems. However, further studies are needed to clarify whether and how synaptic scaling uses GluA1- and GluA2-selective mechanisms and how they might interact.

### Role of GRIP1/PICK1 in synaptic scaling

The GRIP1 and the PICK1 are two PDZ (Post-synaptic density-95/Drosophila disc large tumor suppressor/Zona occludens-1) domain-containing scaffolding proteins that bind the GluA2 subunit at an overlapping site in a competitive manner and control GluA2–AMPAR synaptic accumulation ^43– 45^. While binding to GRIP1 facilitates synaptic accumulation of GluA2 ^46^, PICK1 recruits activated PKC to the receptor complex, leading to phosphorylation of Ser880 on GluA2 that dissociates GRIP1, resulting in AMPAR endocytosis and removal from the synapse ^47– 49^. PICK1 is also known to directly interact with the endocytic machinery, AP2 and dynamin, to facilitate AMPAR endocytosis ^49^. Other studies have suggested that PICK1 is necessary for the intracellular retention of endocytosed AMPARs rather than the initial step of internalization from the cell surface during LTD ^50, 51^. Interaction between GRIP1 and GluA2 is increased following prolonged inactivity in cultured neurons and is essential for surface AMPAR accumulation involved in synaptic upscaling ^40, 41^. Conversely, PICK1 protein level is reduced upon chronic activity blockade but not upon activity elevation ^38^. Inactivity-induced increase in AMPAR mEPSCs is occluded in neurons from PICK1 KO mice because of increased basal levels of synaptic AMPARs, a consequence of enhanced recycling.

### PSD-95

**Figure 1.**
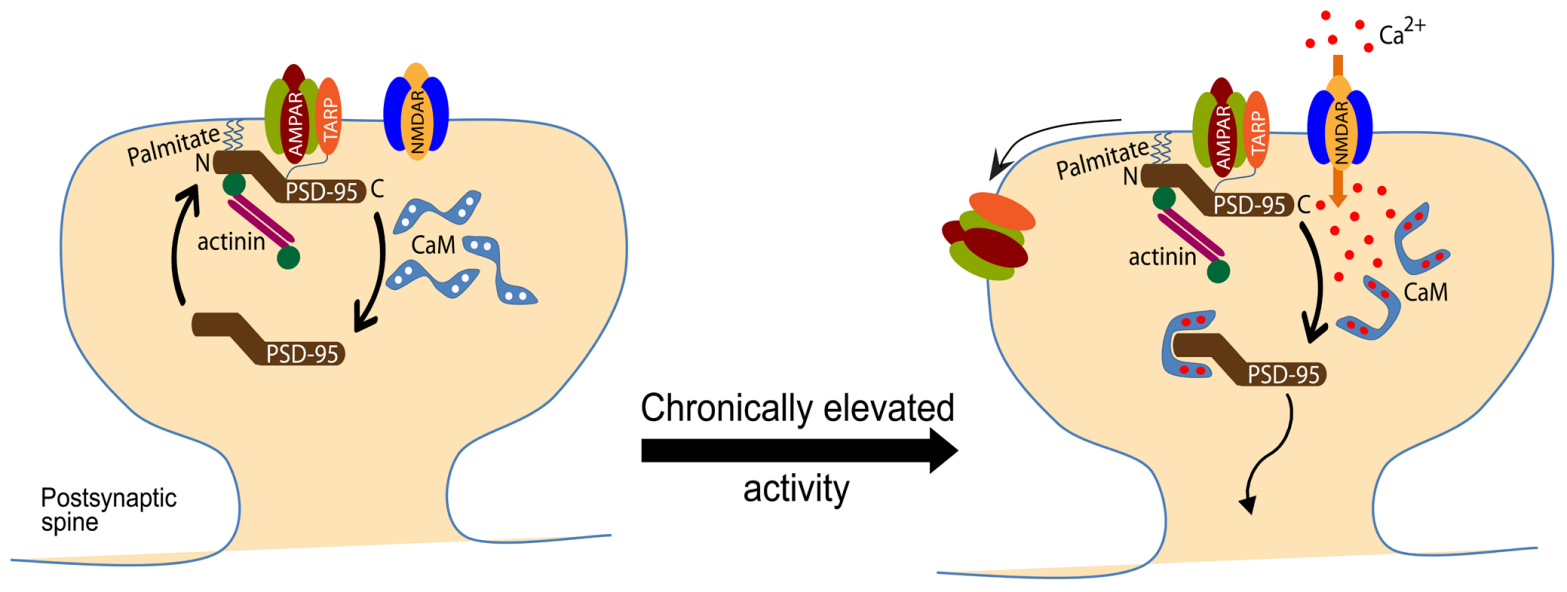
Model for CaM-mediated removal of the synaptic scaffolding protein PSD-95 from excitatory synapses during synaptic downscaling. PSD-95 is targeted to the synaptic plasma membrane through constitutive and fast palmitate cycling at its N-terminus ^58, 71^ and is anchored by α-actinin ^60^. Upon elevation of neuronal activity, calcium influx (Ca ^2+^) through NMDARs leads to an increase in Ca ^2+^/calmodulin (CaM) levels that caps the N-terminus of PSD-95. Such capping of PSD-95 prevents its re-palmitoylation as well as binding to α-actinin and leads to its dispersal from the PSD. With loss of PSD-95, AMPARs become free from being anchored and are subsequently removed from the synapse, resulting in synaptic downscaling ^56^. AMPAR, AMPA-type glutamate receptor; NMDAR, NMDA-type glutamate receptor; TARP, transmembrane AMPAR regulatory protein.

PSD-95, the most abundant post-synaptic scaffolding protein, is a member of the membrane-associated guanylate kinase (MAGUK) family ^52^ and anchors AMPARs at post-synaptic sites by interacting with auxiliary subunits called transmembrane AMPAR regulatory proteins (TARPs) ^53^. Synaptic accumulation of PSD-95 is bidirectionally regulated by chronic activity manipulation and negatively correlates with the direction of perturbation ^54^. Moreover, PSD-95 knockdown blocks both scaling up and down, and PSD-95 overexpression blocks scaling down in cultured neurons, indicating a dominant role for PSD-95 in determining AMPAR synaptic abundance as a function of activity. Such influence of PSD-95 on scaling could be due to its specific role in stabilizing AMPARs at the PSD of mature neurons or a more general role in providing “slots” for the receptors to reside within the PSD. Palmitoylation of PSD-95 at its N-terminal cysteine residues (Cys3 and Cys5) has emerged as a mechanism for activity-dependent regulation of its synaptic localization during scaling ^55, 56^, as would be expected given its absolute requirement for post-synaptic PSD-95 targeting ^57^. Palmitoylation is a common reversible modification regulating membrane insertion of cytosolic proteins. Chronic silencing of activity enhances PSD-95 palmitoylation ^55, 58^ whereas prolonged elevation of activity reduces it ^56^. Such changes are associated with accumulation and loss of PSD-95 from the synapse, respectively. Dynamic recruitment of the PSD-95 palmitoylating enzyme (palmitoyl acyl transferase) DHHC2 to the PSD is induced by activity blockade, which in turn mediates synaptic clustering of PSD-95 and AMPARs ^55^. On the other hand, capping of PSD-95 N-terminus (1–21) by Ca ^2+^/calmodulin (CaM), which sequesters the palmitoylation sites and thereby antagonizes PSD-95 palmitoylation ^59^, leads to PSD-95 dispersal from the PSD and, as a consequence, facilitates AMPAR downscaling ^56^. In the latter study, molecular replacement of endogenous PSD-95 with CaM binding-defective mutants, including the E17R mutation, in cultured hippocampal neurons blocks Bic-induced downscaling of surface GluA1 levels and AMPAR mEPSC amplitude, which is rescued by charge inversion via the coexpression of CaM R126E. Palmitoylation is not sufficient to specifically localize PSD-95 to the post-synaptic site. Recent work identified α-actinin as a critical post-synaptic docking protein for PSD-95 ^60^. Remarkably, α-actinin binds directly to the N-terminus immediately downstream of Cys3 and Cys5. CaM binding to PSD-95 N-terminus not only shifts PSD-95 to a more depalmitoylated state but also displaces it from α-actinin. These findings reveal a precise mechanistic link between increased intracellular Ca ^2+^ levels that trigger downscaling and the homeostatic decrease in synaptic AMPAR levels through CaM-mediated removal of PSD-95 from the synapse ( Figure 1).

### Stargazin

Stargazin (STG) is a TARP member that directly interacts with PSD-95 and dynamically regulates the synaptic stabilization of AMPARs via CaMKII-dependent phosphorylation ^53, 61– 63^. Chronic inactivity increases STG expression and phosphorylation at the CaMKII sites Ser239/240 and Ser228. Overexpression of the phospho-dead mutant of STG prevents TTX-induced increase in synaptic GluA1 levels in cultured neurons. In contrast, overexpression of the phosphomimetic mutant results in higher basal levels of synaptic GluA1 and occludes scaling up ^64^. Additionally, increased STG phosphorylation is observed in the mouse lateral geniculate nucleus concomitant with a parallel reduction in AMPAR rectification index following visual deprivation for 1 week starting at P20, indicating the insertion of CP-AMPARs at those synapses *in vivo*.

Collectively, the data implicating PSD-95 and STG in homeostatic synaptic scaling indicate that the very fundamental mechanism of post-synaptic AMPAR anchoring is affected by neuronal input activity in a manner that substantially contributes to scaling up or down of the post-synaptic AMPAR content.

### Arc/Arg3.1

Activity-regulated cytoskeleton-associated protein (Arc), also known as Arg3.1, is an immediate early gene product that has been implicated in LTP and LTD and in the consolidation of memories ^65^. Strong neuronal activity induces rapid accumulation of Arc at the glutamatergic synapse, where it recruits the clathrin-dependent endocytosis machinery to AMPARs and facilitates their internalization, thus negatively regulating AMPAR-mediated synaptic transmission ^66, 67^. Basal AMPAR–mEPSC amplitudes are increased in Arc KO hippocampal neurons, which fail to show TTX-induced upscaling ^68^. However, Bic-induced downscaling is preserved in these neurons. Moreover, visual experience-induced scaling of AMPAR–mEPSCs in L2/3 principal neurons of primary visual cortex is absent in Arc KO mice, supporting its crucial role in homeostatic regulation *in vivo* ^69^. Briefly, acute re-exposure to light for even 2 hours reversed an increase in mEPSC amplitude induced by 2 days of dark rearing of adult (>P21) wild-type mice, whereas Arc KO mice did not even show changes in mEPSC amplitude upon dark rearing. Recent work has uncovered that the nuclear localization of Arc, apart from its well-established synaptic actions, is required for AMPAR downscaling through repression of GluA1 transcription ^70^. After a transient decrease in nuclear localization at 30 minutes of Bic treatment, Arc shows increased nuclear accumulation at 8 hours of treatment when it regulates GluA1 transcription through cAMP response element (CRE) sites on its promoter. Such a temporal profile fits its fast synaptic role in promoting AMPAR endocytosis to initiate downscaling followed by a long-term reduction in new receptor generation for maintenance of downscaling.

### Homer1a

The immediate early gene product Homer1a has been implicated in the driving of AMPAR downscaling. Under baseline conditions, long-form Homer1 tetramers bind group I metabotropic glutamate receptors (mGluR1/5) and inositol 1,4,5-triphosphate receptors (IP3Rs) and enable glutamate-induced intracellular calcium release. Expression of the short-form monomeric Homer1a is activity-induced and competitively disrupts the scaffolding capabilities of Homer1 to reduce calcium release from intracellular stores ^72^. Homer1a is transiently upregulated by both chronic elevation and silencing of network activity in cultured cortical neurons. Homer1a evokes agonist-independent mGluR activation, which is selectively required for synaptic downscaling ^73^. Genetic ablation of Homer1a results in enhanced surface levels of GluA1 and GluA2 as well as AMPAR-mEPSC amplitude under basal conditions. Both upscaling and downscaling of AMPAR currents are absent in Homer1a KO neurons, although the loss of homeostatic changes in GluA2 surface levels is much more pronounced than that of GluA1. Still, the exact mechanism by which Homer1a mediates AMPAR scaling remains to be explored, although a decrease in GluA2 tyrosine phosphorylation has been suggested to be involved. In a recent study, Homer1a–mGluR signaling was shown to drive global downscaling during sleep in rodents, demonstrating the importance of this signaling in the intact brain ^74^.

### Semaphorin 3F – Neuropilin-2 – PlexinA3 signaling and association with GluA1

Semaphorin 3F was originally identified as an axonal guidance cue. Recent work now implies Semaphorin 3F signaling via its holoreceptor that is formed by the transmembrane proteins Neuropilin-2 and PlexinA3 in downscaling ^75^. Chronic Bic treatment induces Semaphorin 3F secretion and Bic-induced downscaling is absent in dissociated cortical neurons from mice in which Semaphorin 3F, Neuropilin-2, or PlexinA3 is knocked out. Furthermore, Neuropilin-2 interacts with GluA1, and this association is likely mediated by its two extracellular CUB domains with the extracellular N-terminus of GluA1. This interaction is disrupted by Semaphorin 3F and chronic Bic treatment. The resulting downscaling requires the cytosolic C-terminus of PlexinA3 and specifically its Ras GTPase-activating protein (GAP) activity because PlexinA3 with point mutations that impair this activity fails to rescue downscaling in PlexinA3 KO neurons. Defining the role of this Ras GAP activity in downscaling awaits further work, but a hint of how this could work in neurons comes from HEK293 cells expressing this PlexinA3 mutant. In these cells, Semaphorin 3F fails to dissociate GluA1 from Neuropilin-2 in contrast to HEK293 cells expressing wild-type PlexinA3. What is somewhat puzzling is that prolonged (24–48 hours) Semaphorin 3F application induces downregulation of GluA1 surface expression in this work; however, in an earlier study, it leads to an increase in AMPAR–mEPSC amplitude within 2–3 hours in acute rat hippocampal slices ^76^. Perhaps different systems or application conditions result in differential outcome.

## Concluding remarks

The different molecular mechanisms underlying synaptic scaling, as highlighted above, might seem to be independent, but all of them converge onto regulating either trafficking or synapse stabilization of AMPARs. The question is whether all of these molecular events operating at distinct stages of the AMPAR life cycle are essential to achieve scaling or are redundant. Given the multitude of molecular targets recruited in producing long-lasting changes in synaptic strength during Hebbian plasticity (LTP and LTD), we think an effective homeostatic counterbalance might require synergism among at least some of the various scaling mechanisms, each balancing individual perturbed components of the machinery. Considering the shared final molecular output, it is not surprising that both Hebbian plasticity and synaptic scaling involve many of the same molecular targets. This raises the inevitable question of whether and how these two opposing plasticity phenomena (LTP versus downscaling and LTD versus upscaling) that are triggered by similar changes in input activity interact with each other. While acute changes in synaptic activity are sufficient to trigger Hebbian plasticity, homeostatic plasticity is manifested usually upon prolonged perturbations of the same. The much slower operating timescale of homeostatic scaling might be advantageous since compensatory mechanisms that are too quick could disrupt information storage through input-specific associative plasticity. In fact, such temporal segregation of the two forms of plasticity has been suggested to exist in V1 neurons of freely behaving young rats subjected to visual deprivation ^77^, although the mechanistic details remain to be explored. Nonetheless, owing to its control of overall neuronal excitability, homeostatic scaling can impact subsequent induction of Hebbian plasticity in a manner reminiscent of metaplasticity. Recent studies using hippocampal slice cultures found that chronic inactivity affects the LTP induction ability of CA3–CA1 synapses ^78, 79^, although the findings reported are contradictory.

Ever since its discovery, homeostatic synaptic scaling has been studied mostly as a global, cell-wide proportional change in synaptic strength across the majority of synapses on a given neuron. However, it is becoming increasingly evident that a local, synapse-autonomous form of homeostatic regulation also exists to enable input-specific tuning of a restricted set of synapses. In an elegant study by Béïque *et al*., two-photon glutamate uncaging onto synapses apposed to sparse pre-synaptic terminals, which were functionally suppressed, revealed larger AMPAR-mediated currents in “silenced” synapses compared with neighboring synapses receiving unsuppressed pre-synaptic inputs, thus establishing the phenomenon of synapse-specific homeostasis ^29^. *In vivo* evidence for this form of homeostasis was found in the neurons of the optic tectum of Xenopus tadpoles which receive segregated afferents from both visual and mechanosensory pathways ^80^. It was demonstrated that prolonged manipulation of sensory stimulation of each pathway resulted in homeostatic modification exclusively at synapses of the same pathway. Mechanistically, expression of synapse-autonomous homeostasis involves the insertion of GluA2-lacking AMPARs and depends on Arc, indicating some extent of convergence between local and cell-wide forms of homeostasis. As further cellular and molecular details of both cell-wide and synapse-specific forms of homeostatic plasticity will be uncovered, we expect the emergence of new insights into the possible functions of each phenomenon and their interaction within the broader context of neuronal homeostasis. Overall, despite immense advances in the past decade, the field of synaptic homeostasis is relatively new and requires further exploration to fully appreciate its fundamental role in stable information processing in the brain.
